# Low-Temperature Electrospinning-Fabricated Three-Dimensional Nanofiber Scaffolds for Skin Substitutes

**DOI:** 10.3390/mi16050552

**Published:** 2025-04-30

**Authors:** Qiqi Dai, Huazhen Liu, Wenbin Sun, Yi Zhang, Weihuang Cai, Chunxiang Lu, Kaidi Luo, Yuanyuan Liu, Yeping Wang

**Affiliations:** 1 The Third Affiliated Hospital of Shanghai University, Wenzhou 325000, China; qqidia@163.com; 2 School of Medicine, Shanghai University, Shanghai 200444, China; lesyinz@163.com (H.L.); 1849893451@shu.edu.cn (K.L.); 3 School of Mechatronic Engineering and Automation, Shanghai University, Shanghai 200444, Chinazhangyishu@shu.edu.cn (Y.Z.); cxlu@shu.edu.cn (C.L.); 4 Wenzhou People’s Hospital, Wenzhou 325000, China; 5 Department of Obstetrics and Gynecology, The Third Clinical Institute Affiliated to Wenzhou Medical University, Wenzhou 325000, China

**Keywords:** mussel adhesive protein, low-temperature electrospinning, three-dimensional, nanofiber scaffolds, skin substitutes

## Abstract

Severe skin damage poses a significant clinical challenge, as limited availability of skin donors, postoperative skin defects, and scarring often impair skin function. Traditional two-dimensional (2D) nanofibers exhibit small pore sizes that hinder cellular infiltration, unable to simulate the three-dimensional (3D) structure of the skin. To address these issues, we developed 3D porous nanofiber scaffolds composed of polycaprolactone–polylactic acid–mussel adhesive protein (PLGA-PCL-MAP) using low-temperature electrospinning combined with nano-spray technology. Meanwhile, this 3D scaffold features high porosity, enhanced water absorption, and improved air permeability. The incorporation of mussel adhesive protein (MAP) further increased the scaffold’s adhesive properties and biocompatibility. In vitro experiments demonstrated that the 3D nanofiber scaffolds significantly promoted the adhesion, proliferation, and migration of epidermal keratinocytes (HaCaTs) and human fibroblasts (HFBs), while providing ample space for inward cellular growth. Successful co-culture of HaCaT and HFBs within the scaffold revealed key functional outcomes: HaCaTs expressed keratinocyte differentiation markers CK10 and CK14, while HFBs actively secreted extracellular matrix components critical for wound healing, including collagen I, collagen III, and fibronectin. This skin substitute with a composite structure of epidermis and dermis based on three-dimensional nanofiber scaffolds can be used as an ideal skin replacement and is expected to be applied in wound repair in the future.

## 1. Introduction

Skin defects are very common in daily life [1]. When the skin is damaged due to trauma or disease, the difficulty in wound healing and the formation of scars can seriously affect the physical and mental health of patients and bring significant medical and economic burdens [2]. Despite the progress made by traditional skin tissue engineering methods, they are still unable to effectively deal with large-area injuries or produce full-layer grafts [3]. In recent years, 3D skin tissue substitutes have been prepared by electrospinning [4], bioprinting [5], freeze-drying [6], microfluidic chips [7], hydrogels [8], and other techniques. Among them, electrospinning is a promising technology that can produce nanofibrous scaffolds with very high surface area ratios, porous structures [9], and excellent mechanical properties, whose structural and physical properties are similar to those of extracellular matrices (ECMs) [10], as well as the adsorption of wound exudates and enhancement of nutrient and waste transfer [11,12]. Electrospinning nanofiber scaffolds has become a popular research topic for skin tissue engineering.

Conventional electrospinning scaffolds are limited to 2D structures, making it difficult to mimic the human skin structure. Compared to traditional 2D scaffolds, 3D scaffolds provide a better platform for cell-cell interactions, cell migration, and cell morphogenesis, which are important for regulating cell cycle and tissue function [13,14]. With larger pores, high porosity, and a 3.3-fold increase in water absorption compared to 2D membranes, the scaffolds improved cell adhesion, proliferation, and migration and promoted early re-epithelialization and in vivo sarcomeric tissue formation [15]. In recent decades, various methods for preparing 3D nanofiber scaffolds have been reported, such as multilayer electrospinning [16], template-assisted electrospinning [17], porous-doped electrospinning [18], and post-processing electrospinning [19]. Yu et al. [15] prepared 3D nanofiber scaffolds of polycaprolactone-polyethylene glycol-polycaprolactone using a composite process of electrospinning, mechanical cutting, freeze-drying, and heat treatment. Park et al. [20] used electrospinning to prepare novel 3D scaffolds composed of sericin proteins (SF) with the addition of NaCl crystals and co-cultivated keratin-forming cells with fibroblasts in SF scaffold culture to construct an artificial bilayer skin [20]. However, these methods for preparing porous fiber materials are complex and require a large amount of organic solvents. The pore diameters produced by these methods are uncontrollable and require post-treatment, which limits their practical application.

Poly (lactic acid–glycolic acid) (PLGA) and polycaprolactone (PCL) are polyesters approved by the U.S. Food and Drug Administration [21]. PLGA, as a polymeric material, has good biocompatibility, biodegradability, and high mechanical strength [22]. Therefore, PLGA is widely used in the field of regenerative medicine for wound repair, drug release, and implantable bioscaffolds [23]. PCL is a biodegradable synthetic polymer with a low melting point, good solubility, biocompatibility, stability, and good mechanical properties [24]. PLGA-PCL nanofibers are hydrophobic on the surface, and their lack of cell adhesion sites on the surface and bottom of cell adhesion affects their migration, proliferation, and differentiation, and thus can be overcome by doping with other biomaterials [25]. MAP is derived from marine mussels [26], and MAP has become a widely used bio-glue due to its strong adhesion to bioactive molecules, good biocompatibility with cells, and low toxicity, anti-inflammatory, and antioxidant activities [27,28,29]. We compare the properties of materials commonly used in electrospinning (including synthetic polymers, and biomaterials), as shown in Table S1 of Supplementary Materials [30,31]. Three-dimensional nanofiber scaffolds can make up for the shortcomings of traditional scaffolds, such as poor adhesion, low biocompatibility and mechanical strength, and promote the study of their in vitro scaffold properties for skin tissue engineering applications [32].

In this study, we developed 3D porous nanofiber scaffolds composed of polycaprolactone–polylactic acid–mussel adhesive protein (PLGA-PCL-MAP) using low-temperature electrospinning combined with nano-spray technology. Meanwhile, this 3D scaffold features high porosity, enhanced water absorption, and improved air permeability, which were superior to those of 2D scaffolds. The incorporation of mussel adhesive protein (MAP) further increased the scaffold’s adhesive properties and biocompatibility. We successfully co-cultured HFB and HaCaT cells on 3D nanofiber scaffolds used for preparing double-layer skin substitutes. H&E and Masson staining of HaCaT and HFB cells cultured in 3D scaffolds showed that the cells could effectively penetrate the scaffold, and the immunofluorescent technique revealed that HaCaT cells expressed keratin CK10 and CK14, the signature molecules of epidermal cells. HFB cells successfully secreted collagen I, collagen III, and fibronectin. This skin substitute with a composite structure of epidermis and dermis can be used as an ideal skin replacement and promote wound healing.

## 2. Materials and Methods

### 2.1. Materials

PLGA (lactide/glycolide ratio of 50:50, MW = 100,000 Da), PCL (MW = 80,000 Da), and 1,1,1,3,3,3-Hexafluoroisopropanol (HFIP) solution were purchased from Aladdin (Shanghai, China). Phosphate-buffered saline (PBS), Dulbecco’s modified Eagle’s medium (DMEM), fetal bovine serum (FBS), and penicillin/streptomycin (P/S) were purchased from Solarbio (Shanghai, China). Cell counting kit-8 (CCK-8) reagent was acquired from APExBIO (Beijing, China). Live/dead staining reagents were obtained from Solarbio (Shanghai, China). Phalloidin and 4′,6-diamidino-2-phenylindole (DAPI) were obtained from Solarbio (Shanghai, China). The Masson dye kit was purchased from ASPEN (AS1042), and the H&E staining kit was purchased from Solarbio (Shanghai, China). Mouse anti-cytokeratin 10 (CK10, 1:500 dilutions, Abcam, Cambridge, MA, USA). Mouse anti-cytokeratin 14 (CK14, 1:500 dilutions, Abcam). Anti-collagen Ⅰ (ab21286, 1:200 dilutions, Abcam). Anti-collagen Ⅲ (ab7778, 1:200 dilutions, Abcam). Anti-fibronectin (ab2413, 1:200 dilutions, Abcam). Goat anti-mouse secondary antibody (Thermo Fisher, Waltham, MA, USA). Goat anti-rabbit secondary antibody (Thermo Fisher).

### 2.2. Preparation of PLGA-PCL-MAP 2D Nanofiber Scaffolds

First, a mass ratio of PLGA/PCL (15%:5%) was dissolved in HFIP solution and stirred at 180 rpm for 5 h at room temperature until the PLGA-PCL solution was completely mixed. Configure the MAP solution with 0%, 0.5%, 1%, and 2% mass ratios and add to the above solution. The well-mixed PLGA-PCL-MAP solution was drawn into a 10 mL syringe equipped with a 21 G metal needle. The syringe was mounted on the electrospinning platform. The positive pole of the high-voltage power supply was connected to the metal needle, and the negative pole was connected to the electrospinning receiving platform. The parameters of electrospinning were set as follows: the positive voltage was 11 KV, the negative voltage was 0 KV, the flow rate of the micro-syringe pump was 2 mL/h, and the distance between the metal needle and the electrospinning receiving platform was 15 cm. Electrospinning was performed at room temperature and 25–45% humidity. Finally, the obtained PLGA-PCL-MAP 2D nanofiber scaffolds were oven-dried at 35 °C.

### 2.3. Preparation of PLGA-PCL-MAP 3D Nanofiber Scaffolds

As described above, the configured solution of PLGA/PCL/MAP was aspirated into a 10 mL syringe, and the water reservoir of the nano-spray was filled with deionized water and placed 20 cm from the electrospinning device. We adjusted the voltage to 15 kV, shortened the distance between the needle and the low-temperature receiving plate to about 10 cm, and adjusted the flow rate to 1 mL/h to avoid blockage caused by the increase of solution viscosity at low temperature. When the temperature of the cryogenic cooling device drops to −20 °C, the nano water sprayer is turned on, and the sprayer is aligned with the cryogenic collection plate to ensure that the water vapor is deposited on the surface of the cryogenic collection plate to form ice crystals. After 30 min, the prepared 3D nanofiber scaffold was transferred from the low-temperature cooling plate to the refrigerator at −80 °C, frozen for 2 h, and then added to the Borden Freeze-Dryer to freeze-dry for 12 h.

### 2.4. Characterization of 2D and 3D Nanofiber Scaffolds

#### 2.4.1. Scanning Electron Microscope (SEM)

The samples were sputtered and gold-plated, and the morphology of the 2D and 3D nanofiber scaffolds was determined by scanning electron microscopy (Zeiss Thermal Field Scanning Electron Microscope Gemini SEM 300, Shanghai, China) at an accelerating voltage of 10 kV. The average diameter of the nanofibers was calculated by randomly measuring 100 fibers using the Image-Pro Plus software (Image-Pro Plus 6.0, Silver Spring, MD, USA).

#### 2.4.2. FTIR Test

The chemical composition of the nanofiber membranes was evaluated using a spectrometer instrument (NicoletiS10, Thermo Fisher, Waltham, MA, USA). The wavelength range was set from 500 to 4000 cm^−1^ with a resolution of 4 cm^−1^.

#### 2.4.3. Hydrophilicity

To analyze the hydrophilicity of the PLGA-PCL 2D, PLGA-PCL-MAP 2D, and PLGA-PCL-MAP 3D nanofiber scaffolds, measurements were performed using a Dynamic Contact Angle and Permeation Analyzer (BIOLIN Scientific AB, Västra Frölunda, Sweden), which uses a high-resolution, high-speed camera to continuously capture topographical images of the droplets and computer-integrated software to measure the contact angle.

#### 2.4.4. Porosity

The bulk volume of the samples was measured using the ethanol displacement method [33]. The volume percentage of the specimens (2D scaffold: length × width × thickness: 10 mm × 10 mm × 0.2 mm; 3D scaffold: length × width × thickness: 10 mm × 10 mm × 2 mm) was then calculated by dividing the volume of the scaffold at different time intervals by the volume of the initial scaffold. The bulk density of the specimens was calculated by dividing the mass of the specimens by their bulk volume. The porosities of the 2D nanofiber membranes and 3D expanded scaffolds were measured using the liquid displacement method and calculated using Equation (1), written as follows:

(1) $$P\left(\%\right)=\frac{W_{S}\left(g\right)-W_{d}\left(g\right)}{r\left(\frac{g}{{cm}^{3}}\right)\times V\left({cm}^{3}\right)}\times 100\%$$

where *P* is the porosity; W_S_ is the weight of the sample after soaking in ethanol for up to 10 min with a density of ‘*r*’; W_d_ is the weight of the dry scaffold; and *V* is the volume of the sample (*n* = 3).

#### 2.4.5. Water Absorption

The water absorption capacities of different samples were determined based on previous reports. Two-dimensional scaffolds (length × width × thickness: 10 mm × 10 mm × 0.2 mm) and 3D scaffolds (length × width × thickness: 10 mm × 10 mm × 2 mm) with known dry weights (W_d_) were added to 25 mL flasks containing 20 mL of phosphate-buffered saline (PBS, pH = 7.4) solution at room temperature for 2, 3, 4, 5, and 6 min. Excess water was removed from the surface of the sample with filter paper, and the wet weight (W_w_) of the support was recorded again.

Water absorption (w) is calculated according to the following formula:

(2) $$W=\frac{Ww-Wd}{Wd}\times 100\%$$

where *W* is water absorption, W_w_ is the weight after absorption, and W_d_ is the weight in the dry state.

#### 2.4.6. Tensile Test

The mechanical properties of the prepared 2D and 3D nanofiber scaffolds under tension were determined using a Cell Scale, DMA 2980. Canada.The 2D and 3D nanofiber scaffolds were cut into rectangular shapes (length × width: 20 mm × 5 mm), and the original thickness of the scaffolds was measured. Tensile stress tests were performed at room temperature, and the data were analyzed using OriginPro21 software.

### 2.5. Cell Preparation and Biological Characterization

#### 2.5.1. Cell Culture

HaCaT and HFB cell lines were purchased from Fuheng Biotechnology (Shanghai, China). The cells were then incubated at 37 °C and 5% CO_2_. The cells were incubated in Dulbecco’s modified Eagle’s medium (DMEM; Gibco, San Francisco, CA, USA) supplemented with 10% (*v*/*v*) fetal bovine serum (FBS; Gibco) and 1% (*v*/*v*) penicillin/streptomycin (Gibco). When the confluence rate of the cells reached approximately 80%, the cells were passaged in a 1:3 ratio. The prepared nanofiber membranes were cut into 14 mm diameter discs and placed in 24-well plates. Subsequently, the front and back of the nanofiber membrane were sterilized with UV light for 1 h. Before inoculating the membranes with cells, the membranes were rinsed three times with PBS and immersed in DMEM for overnight incubation.

#### 2.5.2. Cell Viability Stain

The nanofiber scaffolds were cut into circles (14 mm in diameter) in 24-well plates and sterilized using UV light for more than 30 min. Cell viability was assessed at a density of 2 × 10^4^ cells/cm^2^. HaCaT and HFB cells were inoculated into 2D and 3D nanofiber scaffolds for 48 h (n = 3). Live/dead staining was used to label the live and dead cells. Prior to the experiments, 2 mM Calcein AM (1.5 μL) and 1.5 mM propidium iodide PI (4 μL) were dissolved in 1 mL of phosphate-buffered saline (PBS, Gibco), according to the instruction manual (Beijing, China, Solarbio), to prepare the experimental solutions. The samples were then removed from the cell incubator and washed gently with PBS. The live staining solution was then added and incubated in a cell culture incubator for 25 min. Subsequently, the dead staining solution was added and incubated for 5 min at room temperature in a dark environment. Finally, the samples were washed with phosphate-buffered saline (PBS). The stained cells were observed under a fluorescence microscope (Tokyo, Japan, Nikon).

#### 2.5.3. Cell Proliferation

The nanofiber scaffolds were cut into circles (14 mm in diameter) in 24-well plates and sterilized using UV light for more than 30 min. To assess cellular value addition, HaCaT and HFB cells (2 × 10^4^ cells/cm^2^) were inoculated onto PLGA-PCL 2D and PLGA-PCL-MAP 2D and 3D nanofiber scaffolds and cultured for 1 day, 3 days, and 5 days. A counting kit-8 (CCK-8, Kumamoto, Japan, Dojindo) was used to detect the proliferation of HaCaT and HFB cells on different scaffolds. The volume ratio of CCK-8 reagent–DMEM solution was 10:100 (*v*/*v*). The prepared mixture was added to a 24-well plate and incubated at 37 °C for 3 h before 100 µL of the solution was pipetted from each sample well and transferred to a 96-well plate (New York, NY, USA, Corning). A 96-well plate was placed in the test area of the enzyme marker (Männedorf, Switzerland, Tecan) to measure the absorbance at an optical density (OD) of 450 nm (n = 3).

#### 2.5.4. Cell Morphology

HFB cells (2.0 × 10^4^ cells/cm^2^) were cultured on 3D nanofiber scaffolds for 48 h. After removing the medium, the cells were washed thrice with PBS and fixed with 4% paraformaldehyde (PFA) for up to 30 min at room temperature. The scaffolds were then washed three times with PBS and immersed in permeabilization solution (0.5% Tritonx-100 (Beijing, China) in PBS solution) for 30 min and closure buffer (1% bovine serum albumin in PBS solution) for 1 h. Subsequently, 5 μg of rhodamine-labeled phalloidin (St. Louis, MO, USA, Sigma) and DAPI (Sigma) solution were used to stain the cytoskeleton and cell nuclei, respectively, in a dark room. Photographs were obtained using a confocal laser-scanning microscope (Carl Zeiss, LSM900, Oberkochen, Germany).

#### 2.5.5. Histology and Immunofluorescence Staining

For histomorphometric analysis, cells were cultured on 3D nanofiber scaffolds for 7 days. Cells were fixed with 4% paraformaldehyde (PFA, Shanghai, China, Beyotime) for 20 min at room temperature and embedded in OCT. Sections of 8 μm thickness were cut with a cryosectioner. Samples were stained with hematoxylin and eosin (H&E, Solarbio, n = 3) Masson’s trichrome. For immunofluorescence analysis, samples were first embedded and blocked with PBS containing 10% sheep serum for 1 h at room temperature or overnight at 4 °C. Samples were rinsed three times for 5 min each with PBS and incubated with the specific primary antibody at 37 °C for half an hour or at room temperature for more than 1 h or overnight at 4 °C. Samples were rinsed three times with PBS for 5 min each time at room temperature. Incubate with secondary antibody at 37 °C for more than 30 min or at room temperature for more than 1 h. Remove the secondary antibody and add DAPI. Rinse three times with PBS at room temperature for 5 min each time. Observe under a fluorescence microscope with appropriate wavelength excitation to obtain a histologic image for evaluation of epidermal cell markers.

## 3. Results

### 3.1. Preparation and Characterization of 3D Nanofiber Scaffolds

#### 3.1.1. Preparation of 3D Nanofiber Scaffolds

The concentration of PLGA-PCL (15%:5%) for electrospinning was as previously reported by the subject [23]. With the increase of MAP concentration, the larger the liquid viscosity, the smaller the nanofiber diameter, as shown in Figure S1. Morphological observation of cells on two-dimensional nanofiber scaffolds containing 0%MAP, 0.5%MAP, 1%MAP, and 2%MAP, HFB cells had poor adhesion morphology on 2D nanofibers without MAP, and the overall spread area of cells was small, and the cell membrane surface showed structural characteristics of local depression or wrapped fibers (Figure S2). The diffusion area of HFB cells on the four membranes was quantitatively analyzed, and the cell adhesion increased with the increase of MAP concentration. HFB cells in PLGA-PCL-1%MAP, PLGA-PCL-2%MAP 2D nanofiber surface morphology is relatively stretched, showing slender filamentous pseudopodia and extending in the direction of the nanofiber arrangement. To avoid the phenomenon of needle clogging due to large MAP viscosity during the experiment, PLGA-PCL-1%MAP concentration was selected as the basis for subsequent experiments.

**Figure 1:**
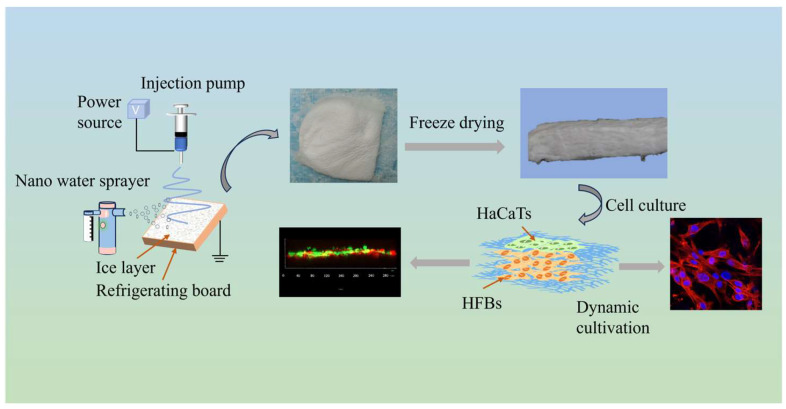
Schematic diagram of the preparation process of 3D nanofiber scaffolds.

**Figure 2:**
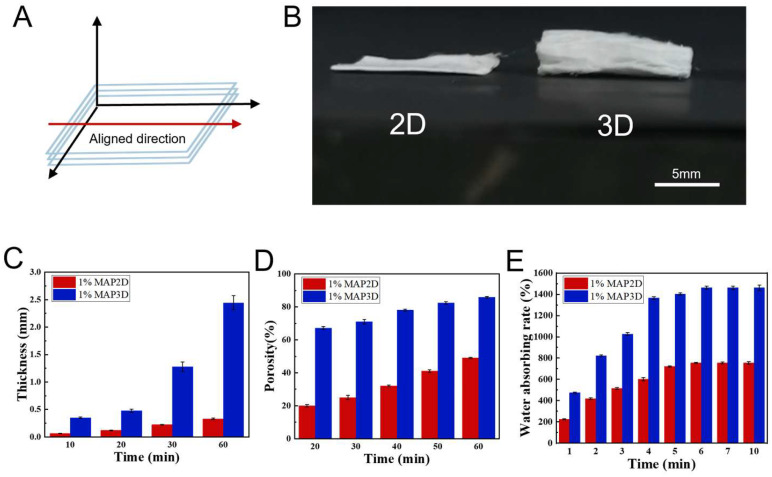
Extension and characterization of 2D and 3D scaffolds. (**A**) The diagram shows the alignment direction of the scaffolds. (**B**) Overall morphology after 30 min of 2D and 3D electrospinning (scale bar = 5 μm). (**C**) Thickness of 2D and 3D scaffolds at different electrospinning times. (**D**) Porosity of 2D and 3D scaffolds at different electrospinning times. (**E**) Water absorption of 2D and 3D scaffolds soaked in PBS solution.

The fabrication scheme of the 3D scaffold is shown in Figure 1. When the temperature of the receiving plate drops below 0 °C, a thin layer of ice crystals is formed on the surface of the receiving plate by the solidification of water vapor sprayed by the nano-sprayer, and the lower the temperature of the receiving plate, the denser the ice crystals. The deposition of fibers and the formation of ice crystals occurred simultaneously during the experiment, and the electrospinning nanofibers froze and self-assembled on the ice crystals to form 3D frozen nanofiber membranes with a certain thickness when they encountered the collector plate below 0°, which were then lyophilized in a freezing dryer to produce porous scaffolds (Figure S3). Figure 2A shows a schematic of the scaffold orientation direction; the red arrows indicate the fiber orientation direction, and relatively uniform 3D porous nanofiber scaffolds were prepared after optimizing the electrospinning parameters to avoid the formation of beaded nanofibers. Figure 2B shows a photograph of 2D and 3D scaffolds after half an hour of electrospinning. In Figure 2C, the thickness of 2D and 3D scaffolds increased with increasing electrospinning time, but the thickness of 3D scaffolds increased more rapidly, which was mainly due to the nucleation of cryogenic receiving plates and ice crystals, which allowed the nanofibers to extend the 3D structure with increased porosity. In Figure 2D, the porosity of 3D scaffolds increases from 67.2% ± 1.92 to 85.8% ± 1.30 with increasing electrospinning time, which is much larger than that of 2D scaffolds. In Figure 2E, the water absorption capacity of 3D scaffolds was much stronger than that of 2D scaffolds, and both 2D and 3D scaffolds reached the equilibrium absorption capacity at 6 min.

#### 3.1.2. Microstructure

**Figure 3:**
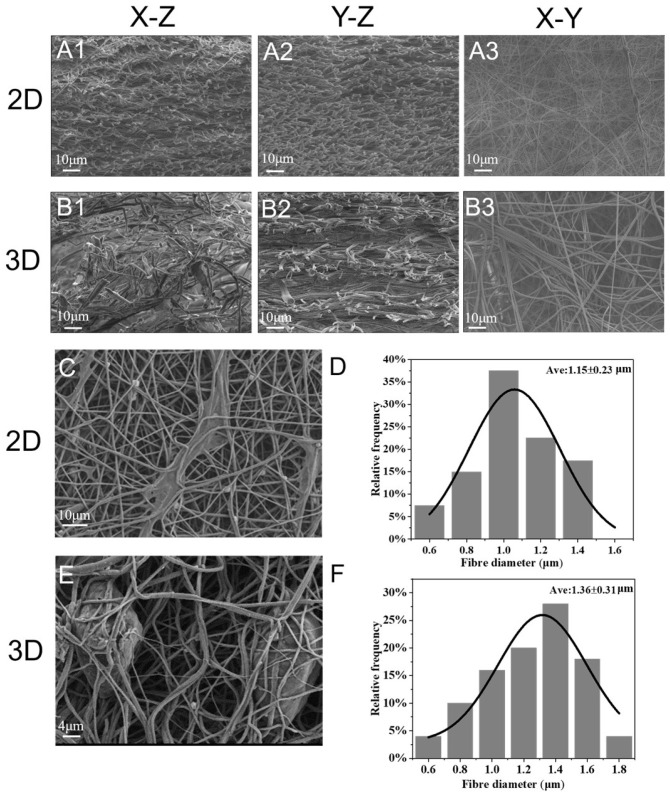
Scanning electron microscope images of 2D and 3D scaffolds: (**A1**–**A3**) Cross-sectional morphology of 2D scaffolds (scale bar = 10 μm). (**B1**–**B3**) Cross-sectional morphology of 3D scaffolds (scale bar = 10 μm). (**C**) SEM scanning morphology of HFBs on 2D scaffolds (scale bar = 10 μm). (**D**) Diameter distribution of 2D scaffolds. (**E**) SEM scanning morphology of HFBs on 3D scaffolds (scale bar = 4 μm). (**F**) Diameter distribution of 3D scaffolds.

SEM analysis was performed to reveal the detailed morphological structures of the 2D and 3D scaffolds. The cross-section of the 2D scaffolds consisted of densely arranged fiber structures, Figure 3(A1–A3), and the cross-section of the 3D scaffolds had a continuous laminar porous structure with laminar fiber layers (Figure 3(B1–B3)). The distances between the layers of the 3D scaffolds were larger than the distances between the 2D scaffolds, resulting in the formation of loosely woven nanofibers. Figure 3C, Further observation of cell-scaffold interactions on day 3 by SEM revealed that HFB cells adhered to and spread on the surface of 2D scaffolds and proliferated along the nanofibers. Compared with 2D scaffolds, 3D scaffolds showed a larger pore distribution, and the pores in the network promoted cell infiltration into the scaffolds (Figure 3E). Measured with Image-Pro Plus 6.0 software, the average diameter of the 2D scaffolds was 1.15 ± 0.23 um and that of the 3D scaffolds was 1.36 ± 0.31 um. The presence and density of ice crystals are likely to affect the alignment and orientation of the fibers, leading to differences in macroscopic morphology (Figure 3D,F). The 3D scaffolds showed more cell infiltration, indicating that the cells were more likely to grow toward the inside of the 3D scaffolds. The cells adhered to the nanofibers and proliferated along the nanofiber network with a normal phenotype and elongated morphology. The 3D scaffolds exhibited a hierarchical and porous structure, which would be beneficial for nutrient exchange and intercellular communication [34].

#### 3.1.3. Characterization of 3D Nanofiber Scaffolds

**Figure 4:**
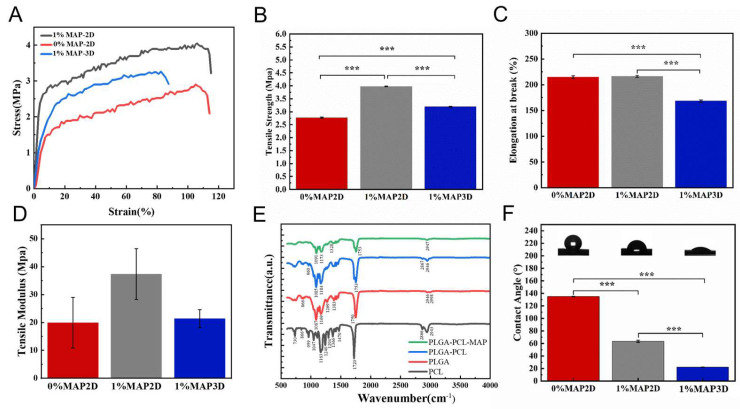
(**A**) Stress–strain curves. (**B**) Tensile strength. (**C**) Elongation at break. (**D**) Tensile modulus. (**E**) FTIR spectra of different scaffolds. (**F**) Water contact angle of different scaffolds (*** *p* < 0.001).

The preparation of 3D nanofiber scaffolds via electrospinning remains challenging, with most products reported in the literature being cotton-like nanofiber deposits that exhibit poor mechanical properties [35]. The mechanical properties of the nanofiber scaffolds significantly affect cell proliferation, migration, and differentiation [36]. The prepared 3D nanofiber scaffolds exhibited excellent mechanical properties and elastic modulus. We compared 2D nanofiber scaffolds without MAP to those with MAP, and the results showed that the stress–strain of the nanofiber scaffolds increased from 2.85 ± 0.03 MPa to 3.97 ± 0.04 MPa, the elastic modulus from 23.13 ± 6.70 MPa to 43.55 ± 16.24 MPa, and the tensile strength from 2.77 ± 0.07 MPa to 3.77 ± 0.07 MPa, after the addition of MAP. With no significant difference in elongation at break. This is mainly due to the addition of MAP, which increases the viscosity of the liquid. The 3,4-dihydroxyphenylalanine (DOPA) groups in MAP are coupled to the ends or side chains of PLGA or PCL polymers, which can form a crosslinked spatial site-resistive network, and the Young’s modulus and ultimate tensile strength of the nanofibers were significantly increased. We then tested the mechanical properties of the 2D and 3D nanoscaffolds, with the 2D scaffolds having an ultra-low packing density and the 3D scaffolds having a relatively fluffy structure with a stress–strain reduction of 3.23 ± 0.01 MPa, along with a relatively low Young’s modulus of 22.42 ± 7.04 MPa, tensile strength of 3.19 ± 0.04 MPa, and elongation at break (Figure 4A–D). Good mechanical properties and stretch resistance enable the 3D nanofiber scaffold to maximize the protection of the damaged skin under external stress [37,38]. Good elasticity provides a 3D dynamic microenvironment; therefore, 3D nanofiber scaffolds can effectively regulate cell behavior [33].

In the FTIR spectra [39,40] (Figure 4E), the main characteristic peaks of PCL were observed at 3000 cm^−1^ (asymmetric CH_2_ stretching), 2850 cm^−1^ (symmetric CH_2_ stretching), 1724 cm^−1^ (carbonyl stretching), 1293 cm^−1^ carbonyl group (C=O) and C=C stretching, and 1169 cm^−1^ (symmetric C-O-C stretching). These characteristic bands agree with those of previous studies; the characteristic peaks of PLGA, such as those observed between 1750 cm^−1^ and 1760 cm^−1^, represent carbonyl (C=O) stretching vibrations, and the peaks between 1200 cm^−1^ and 1250 cm^−1^ represent C=O (reductive) stretching vibrations. The peaks between 1200 cm^−1^ and 1250 cm^−1^ represent C=O (due to the stretching of the ester group). In the PLGA-PCL-MAP spectra, new characteristic absorption peaks appeared at 1556 cm^−1^ and 3350 cm^−1^, which are attributed to the stretching vibrations of -NH_2_ and the benzene ring [32,41]. This characteristic peak proves that a composite nanofiber scaffold containing MAP was successfully prepared.

Changes in the surface wettability of the different scaffolds were analyzed using the water contact angle. Figure 4F shows that the stent without MAP has poor hydrophilicity and a water contact angle of 135°. The contact angle of the scaffolds containing MAP is 63°, and the hydrophilic surface of the scaffolds containing MAP is better than that without MAP because MAP contains a large number of hydrophilic groups, such as amines or hydroxyl groups, which can be combined with hydrophobic materials to form hydrogen bonds or covalent bonds [42]. This increases the roughness and hydrophilicity of the nanofiber surface, resulting in a lower water contact angle to promote cell proliferation and adhesion [32,43]. The contact angle of the 3D scaffolds decreased further to 22°, which may be related to the undulating microtopography and large voids on the surface of the 3D scaffolds. PLGA and PCL have been widely used as biodegradable and biocompatible polymers in the field of tissue engineering because of their good structural stability and tunable mechanical properties [44,45]. However, its hydrophobic properties limit its cell adhesion and proliferation. MAP, derived from the foot filament threads of marine mussels, has excellent wet-state adhesion capabilities, and the PLGA-PCL-MAP nanofiber scaffolds greatly improve the wettability of the sample surface, thereby promoting cell adhesion and proliferation [46,47].

### 3.2. Three-Dimensional Nanofiber Scaffolds Promote Cell Viability

#### 3.2.1. Promoted Cell Viability 

**Figure 5:**
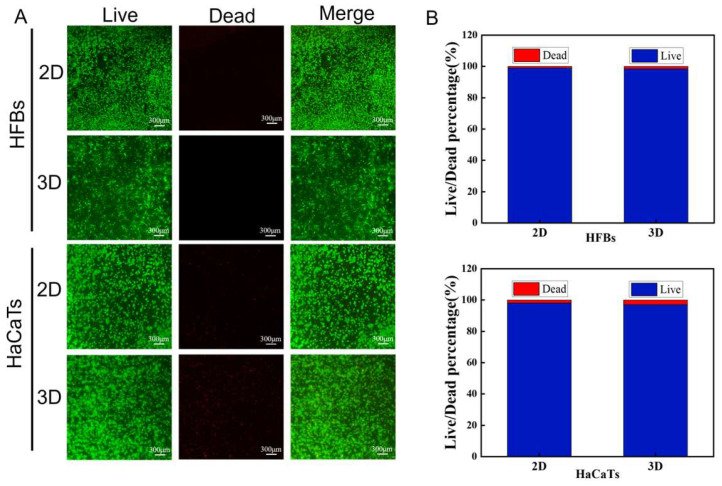
Cell live–dead staining and quantitative analysis: (**A**) Live–dead staining images of HFBs and HaCaTs on 2D and 3D scaffolds (scale bar = 300 μm). (**B**) Quantitative analysis of live–dead cells on 2D and 3D scaffolds of HFBs and HaCaTs.

To study cell viability on the scaffolds, HFB and HaCaT cells were inoculated on 2D and 3D nanofiber scaffolds, and after 48 h of incubation, specifically labeled bright green fluorescence for live cells and red for dead cells, and live–dead cell staining indicated that the HFB and HaCaT cells had high cell viability, as shown in Figure 5A. Figure 5B shows quantitative analysis of live and dead cells of HFBs and HaCaTs on 2D and 3D scaffolds; the PLGA-PCL-MAP nanofiber scaffolds had good cytocompatibility.

#### 3.2.2. Culture and Proliferation of Dermis

**Figure 6:**
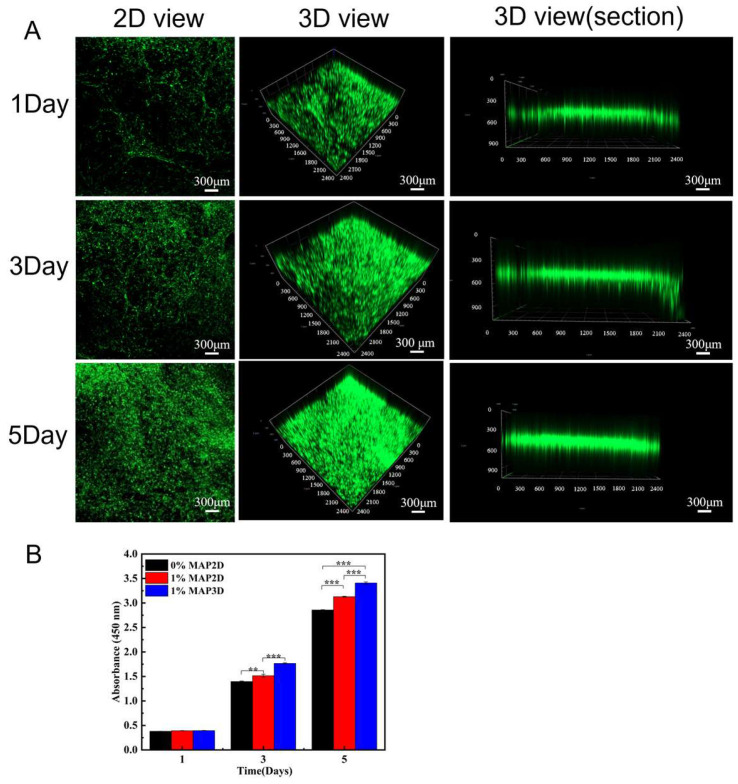
(**A**) Confocal pictures of HFB cells cultured on 3D scaffolds for 1, 3, and 5 days (scale bar = 300 μm). (**B**) CCK-8 assay of HFB cells cultured on PLGA-PCL 2D, PLGA-PCL-MAP 2D and 3D scaffolds for 1, 3, and 5 days (*** *p* < 0.001, ** *p* < 0.01,).

The CCK-8 assay for cell proliferation on days 1, 3, and 5 was performed to assess whether the 2D and 3D scaffolds were suitable for HFB cell adhesion and proliferation. CCK-8 results showed that HFB cells were present in 2D scaffolds without MAP and with MAP and 3D nanofibrous scaffolds, indicating that the three groups of scaffolds had good biocompatibility. Figure 6B showed that after 3 days of culture, HFB cells had a higher proliferation and adhesion ability on both 2D and 3D scaffolds with MAP than on 2D scaffolds without MAP, which may be attributed to the strong adhesion of the MAP in the moist environment. In addition, with an increase in culture time, HFB cells inoculated on 3D scaffolds maintained a high proliferation rate compared to those on 2D scaffolds. This may be attributed to the 3D layered nanofiber structure, which provides a wider surface area and porosity for cell growth and infiltration, and the three-dimensional porous structure provides sufficient space for inward cell proliferation and migration [48,49,50]. Confocal images of HFB cells grown on 3D scaffolds for 1, 3, and 5 days (Figure 6A). Three-dimensional scaffolds showed desirable biocompatibility, with stronger cell-3D scaffold interactions, and provided the microenvironment required for cell growth and proliferation, which was more conducive to repairing damaged tissues.

#### 3.2.3. Culture and Proliferation of the Epidermis

**Figure 7:**
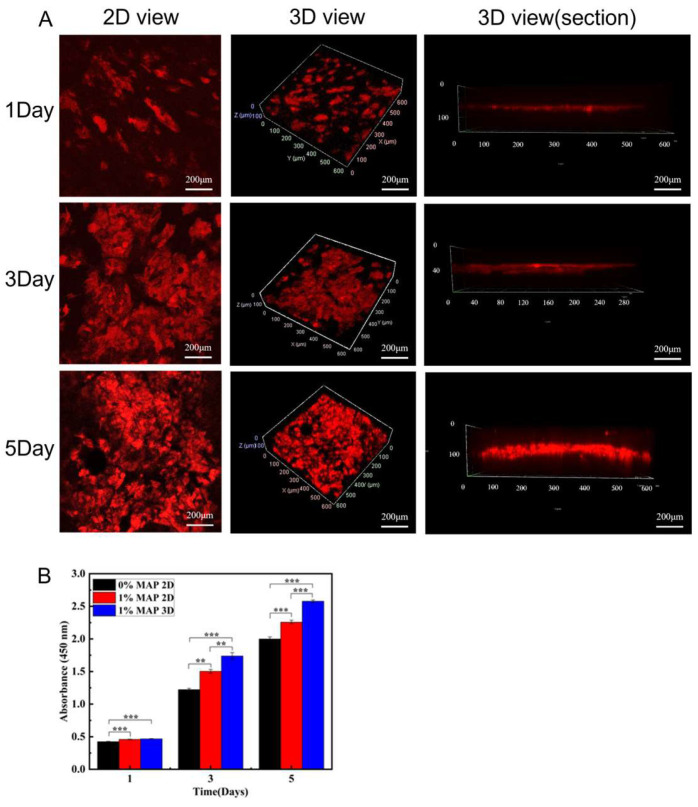
(**A**) Confocal images of HaCaT cells cultured on 3D scaffolds for 1, 3, and 5 days (scale bar = 200 μm). (**B**) Proliferation data of HaCaT cells cultured on PLGA-PCL 2D and PLGA-PCL-MAP 2D and 3D scaffolds for 1, 3, and 5 days (*** *p* < 0.001, ** *p* < 0.01).

Using the vertical view of the confocal images, we investigated the cellular penetration depth of HaCaTs during culture. On the 3D scaffolds, the penetration depth of HaCaT cells increased from 50 μm on day 1 to 140 μm on day 5 (Figure 7A). Cell penetration is critical for 3D tissue regeneration, and most polymer scaffolds are hydrophobic, so cells can only penetrate into the inner regions by degrading the material, which is a rather slow process [51]. We similarly detected the proliferation ability of HaCaT cells on days 1, 3, and 5 using CCK-8 to assess whether the 3D scaffolds were suitable for HaCaT cell adhesion and proliferation. The results showed that the proliferation of HaCaT cells on 2D and 3D scaffolds with MAP was higher than that on 2D scaffolds without MAP, and the strong adhesion of MAP promoted the cell adhesion and proliferation. Similarly, HaCaT cells grew well on 3D scaffolds, and their proliferation was higher than that on 2D scaffolds (Figure 7B). The average fluorescence intensity of HaCaT and HFB cells cultured on a 3D scaffolds 1, 3, and 5 were supplemented in Figure S4. This was mainly due to the super-hydrophilic and highly porous structure of the 3D scaffolds. These results indicate that the materials and fabrication process are non-toxic to cell growth and that 3D scaffolds favor cell adhesion and spreading.

### 3.3. Construction of Skin Substitutes with a Double-Layer Structure of Composite Epidermis and Dermis

#### 3.3.1. Construction of Skin Substitutes

**Figure 8:**
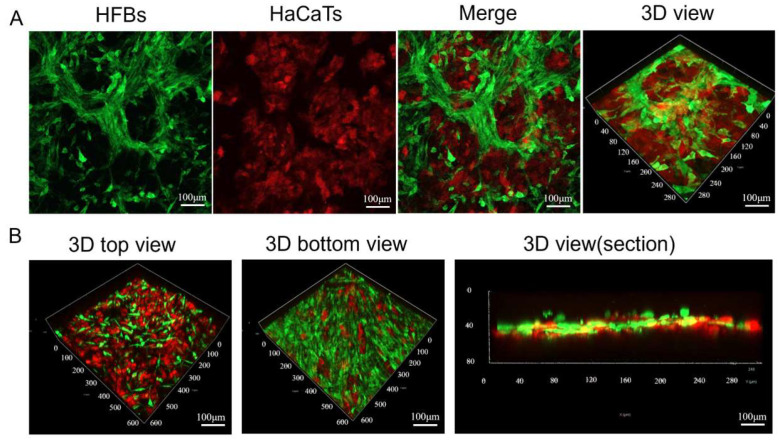
(**A**) Confocal images of HFB and HaCaT cells co-cultured in 3D scaffolds as well as 3D views (scale bar = 100 μm). (**B**) Confocal images of the upper distribution of HaCaT cells in 3D scaffolds and the lower distribution of HFB cells in 3D scaffolds, as well as 3D cross-sectional views of their co-cultures (scale bar = 100 μm).

We continued to study the co-culture of HFB and HaCaT cells on the 3D nanofiber scaffolds. First, we inoculated HaCaT cells with red fluorescent labeling at a concentration of 2 × 10^4^ cells/cm^2^ in the upper layer of the 3D nanofiber scaffolds and cultured them for more than 4 h to make the HaCaT cells adhere to the scaffolds, and then HFB cells with green fluorescent labeling at a concentration of 2 × 10^4^ cells/cm^2^ were inoculated in the lower layer of the 3D nanofiber scaffolds and cultured together for 48 h. Observation under a confocal microscope showed the layered structure of the epidermis and dermis produced by co-culture of HaCaT and HFB cells, respectively. Figure 8A shows images of HFB and HaCaT cells co-cultured on 3D nanofiber scaffolds, as well as 3D views. Figure 8B shows the top view of HaCaT cells cultured on 3D nanofiber scaffolds, the bottom view of HFB cells, and a 3D cross-sectional view. We successfully co-cultured keratinocytes with fibroblasts in an in vitro 3D scaffold to generate a bilayer skin substitute in vitro.

#### 3.3.2. Characteristics of Epidermis and Dermis in Skin Substitutes

**Figure 9:**
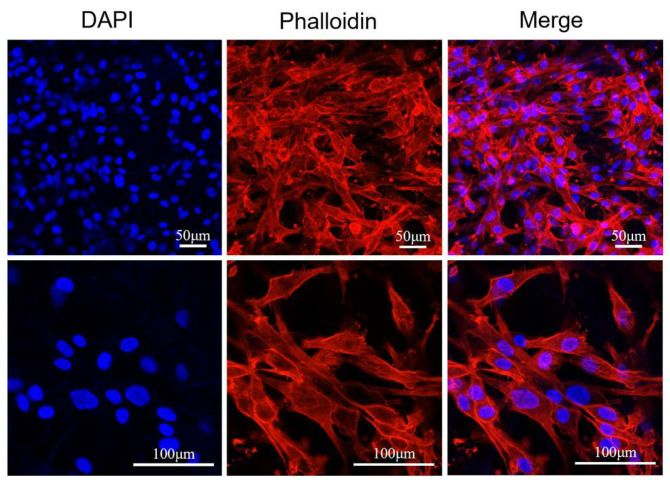
Confocal images of cytoskeleton and nucleus staining of HFB cells (scale bar = 50 μm, 100 μm).

Phalloidin/DAPI staining was used to study the morphology of HFB cells on 3D scaffolds to further investigate the interaction between cell growth and the 3D nanofiber scaffolds. HFB cells showed good adhesion and growth on 3D nanofiber scaffolds, and the cytoskeleton had a diffuse morphology, with more dispersed cells and larger nuclei after 48 h of culture. This indicates that owing to the large pore size and loosely stacked structure of the 3D scaffolds, the cells infiltrated the 3D scaffolds, thus providing more space for cell spreading (Figure 9). A three-dimensional honeycomb porous structure that provides sufficient oxygen and nutrient exchange to better support cell growth is often considered the main challenge in culturing cells in 3D scaffolds.

**Figure 10:**
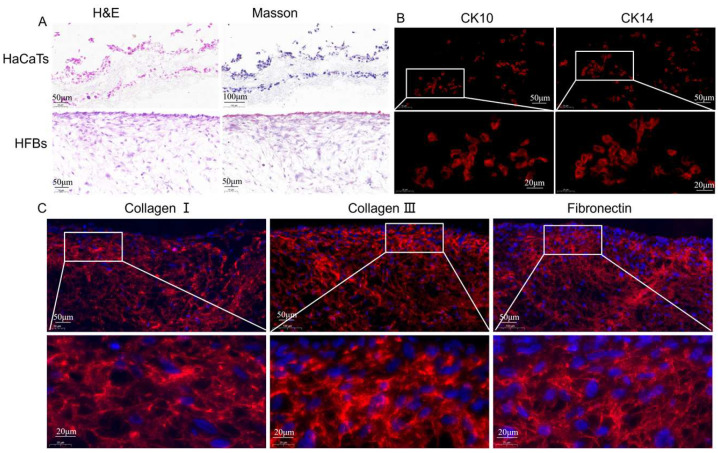
(**A**) H&E and Masson trichrome staining. (**B**) Immunofluorescence of ck10 and ck14. (**C**) Immunofluorescence staining of collagen I, collagen III, and fibronectin (scale bar = 20 μm, 50 μm, 100 μm).

We immobilized HaCaT and HFB cells on 3D nanofiber scaffolds, performed frozen sections, and observed cell growth within the fiber membrane and in the cross section by H&E and Masson staining. As shown in Figure 10A, we found that the 3D honeycomb structure contained a wide range of cell distribution in the thickness direction, especially its three-dimensional structure in the z-axis direction, which could provide more adhesion sites for cell proliferation. This result is consistent with the results of immunofluorescence staining that the 3D honeycomb membrane supports cell proliferation in the thickness direction and is characterized by spatial growth. H&E and Masson’s staining showed that HaCaT and HFB cells infiltrated into the interior of the 3D nanofiber scaffold (Figure S5A).

Keratinocytes are in the outermost epidermal layer of the skin and are important component cells that form the barrier between the skin and the outside world. CK10 and CK14 are expressed in epidermal cells and are marker genes for keratinocytes [52]. CK10 is a marker of early differentiation of the epidermis and is mainly found in the stratum spinosum and stratum granulosum [53]. We cultured HaCaT and HFB cells in 3D nanofiber scaffolds for 7 days and detected keratinocyte protein expression by immunofluorescence. As shown in Figure 10B, the results showed that HaCaT cells successfully expressed keratinocyte marker proteins CK10 and CK14 in 3D nanofiber scaffolds. Collagen I is the most abundant fibrous structural protein in ECM. In the process of tissue repair, deposition of collagen I is usually associated with mature scar formation and long-term matrix stability, while collagen III is often co-expressed with collagen I but is more significantly distributed in embryonic development or early wound healing stages. An increased proportion of collagen III is often associated with increased tissue flexibility and fibrosis inhibition [54]. Figure 10C and Figure S5B show that HFB cells successfully expressed collagen I and collagen III in the 3D scaffold, forming a highly ordered fiber bundle network, indicating that the 3D scaffold has the ability to provide mechanical support and tissue tensile strength regulation, and the formed ECM microenvironment has high plasticity and elastic adaptability. Fibronectin is a multifunctional glycoprotein that mediates cell-matrix adhesion through its RGD (Arg-Gly-Asp) domain and integrates the ECM with the cytoskeleton (e.g., through integrin receptors) [55]. The successful secretion of fibronectin by HFB cells suggests the possible formation of functional adhesion patches (focal adhesion) in the scaffolds, and the transient high expression of fibronectin is a marker of the initiation phase of tissue repair and may also promote the maturation of the scaffold’s three-dimensional structure through the recruitment of other ECM components (e.g., collagen protofibril assembly).

## 4. Discussion

Engineered skin substitutes represent a prospective approach for the treatment of acute and chronic skin wounds, such as large burns or ulcers, and bilayer skin substitutes, which explore the interactions between epidermal and dermal components, are widely recognized as an effective approach to skin regeneration [56,57]. Electrospinning nanofibers have the potential to promote skin tissue regeneration [58]. PLGA and PCL are biodegradable synthetic polymers with good biocompatibility, stability, and mechanical properties [23]. However, PLGA-PCL nanofibers have a hydrophobic surface, which lacks cell adhesion sites on the surface and cell adhesion bottom. We introduced MAP, which has excellent wet-state adhesion and is enriched with DOPA and lysine, which have antimicrobial, anti-inflammatory, antioxidant, and cell adhesion-promoting properties, which have attracted much attention in the biomedical field [59,60]. We used low-temperature electrospinning, nano-spraying, and freeze-drying to prepare 3D honeycomb nanofiber scaffolds. Due to the low temperature of the receiving plate, many ice crystals fill the voids during fiber deposition, which will occupy a certain amount of space. The ice crystals are removed after the fiber membrane freeze-drying, and the voids they leave behind make the fiber membrane fluffier [61]. The nucleation of ice crystals allows the nanofibers to expand the 3D structure with increased porosity [62].

In our experiment, we found that the hydrophilicity of the 3D nanofiber scaffold was greatly improved, and the contact angle was reduced, which might be attributed to the liquid permeability and surface roughness and infiltration models of the 3D scaffold. Three-dimensional stents typically have higher porosity and a more open 3D network structure, and liquids are more easily infiltrated into the stent under capillary action, reducing the apparent contact angle. However, the 2D membrane fiber layers are closely arranged, with small pores and poor connectivity, and the liquid is easy to keep on the surface, forming a large contact angle. The high roughness and porosity of the 3D structure are consistent with the Cassie–Baxter infiltration model, where the liquid penetrates the pores to form a “mixed contact” that significantly reduces the apparent contact angle. The 2D structure surface is relatively smooth, closer to the Wenzel model the contact angle is dominated by the intrinsic properties of the material; if the material itself is hydrophobic, the contact angle will be larger. Furthermore, we conducted many slicing experiments, which proved that the 3D scaffold obtained by us using the low-temperature electrospinning technology has uniform porosity, as well as repeatability and stability, as shown in Figure S6.

The advantage of low-temperature electrospinning technology to prepare 3D nanofiber scaffolds is that it can achieve efficient assembly of nanofibers under mild conditions (such as low temperature or freezing environment), avoiding the destruction of heat-sensitive biological materials (such as proteins and natural polymers) by high temperatures or organic solvents [63,64]. The multi-stage pore structure and high specific surface area formed at the same time are conducive to cell infiltration, nutrient transport, and metabolic waste discharge [65]. In addition, the technology can precisely regulate the fiber morphology and scaffold topology by regulating spinning parameters such as solution properties, electric field strength, and receiver temperature, thus simulating the physical properties of the natural extracellular matrix [66,67]. Electrospinning shows great potential in the preparation of 3D nanofiber scaffolds, especially for the construction of bionic tissue engineering scaffolds.

Although electrospinning technology has made significant advances in research to develop alternatives to skin tissue engineering, many challenges remain. First, the range of natural biomaterials that can be electrospun is limited, and their properties are inconsistent [68]. Secondly, the structure and properties of the resulting products are not yet comprehensive, and most applications are still in the experimental stage. The practical clinical application of these products is not yet mature. The preparation parameters of electrospinning organic/inorganic composite nanofibers are related to the structure, aggregation mode, synergistic properties, processing, and composite technology of the nanoparticles [69]. In terms of controllable construction of 3D structures, a low-temperature environment may lead to insufficient solvent volatilization during fiber deposition, resulting in excessive adhesion between fibers or structural collapse, which restricts the accurate construction of complex 3D geometric shapes [70]. At the same time, compared with traditional 3D printing technology (such as melt deposition and light curing), it has disadvantages in terms of macro-mechanical strength and structural stability. Therefore, its application in regenerative medicine can be expanded through cross-linking, multi-process compounding, or post-processing, as well as through process innovation and integration of interdisciplinary technologies [71]. Finally, how to design different skin tissue models to meet the needs of different skin injury patients and how to produce products in a low-cost and efficient way are still urgent problems to be solved.

## 5. Conclusions

In this study, a novel PLGA-PCL-MAP 3D nanofiber scaffolds was prepared using low-temperature electrospinning technology. These 3D scaffolds have a high porosity, specific surface area and three-dimensional structure, which can promote the activity of HaCaT and HFB cells, including adhesion, migration and proliferation, and shows a good cell morphology. Furthermore, HaCaT and HFBs were co-cultured on a 3D scaffold in vitro to construct a double-layered skin substitutes. HaCaT cells expressed keratin CK10 and CK14 specifically in the 3D scaffolds, reflecting their normal differentiation and barrier function. HFB cells secreted collagen I, collagen III and fibronectin efficiently, maintaining the homeostasis of the extracellular matrix. This skin substitutes with epidermal and dermal structures based on 3D nanofiber scaffolds could highly mimics natural skin in structure and function. It is expected to be applied in skin transplantation in the future and serve as an in vitro platform for reliable pathological research.

## Data Availability

The original data presented in the study are included in the article.

## Supplementary Materials

The following supporting information can be downloaded at: https://www.mdpi.com/article/10.3390/mi16050552/s1, Table S1. Properties of common materials (including polymers, biomaterials) used for electrospinning. Figure S1. (A) Scanning electron microscopy of PLGA-PCL-0%MAP, PLGA-PCL-0.5%MAP, PLGA-PCL-1%MAP, PLGA-PCL-2%MAP 2D nanofiber scaffolds. (B) Diameter distribution of nanofibers with different MAP concentrations. Figure S2. (A) SEM images of HFBs cells inoculated on PLGA-PCL-0%MAP, PLGA-PCL-0.5%MAP, PLGA-PCL-1%MAP, PLGA-PCL-2%MAP nanofiber scaffolds were enlarged by 200, 500 and 1000 times, respectively. (B) Diffusion area of HFBs cells on PLGA-PCL-0%MAP, PLGA-PCL-0.5%MAP, PLGA-PCL-1%MAP, PLGA-PCL-2%MAP nanofiber scaffolds. Figure S3. Macrostructure of 3D nanofibers: (A) Fiber deposition on cryo-receiving plate. (B) 3D frozen nanofiber scaffold. (C) 3D dried nanofiber scaffold. Figure S4. Mean fluorescence intensity of HaCaTs and HFBs cells cultured on 3D nanofiber scaffold for 1, 3 and 5 days. Figure S5. (A) H&E and Masson staining of HFBs cells cultured in vitro on 3D scaffold for one week. (B) Overall images of Col I, Col III, and fibronectin immunofluorescence. Figure S6. Cross-sectional slice images of 3D nanofiber scaffolds.
