# Supplementary material for: Low-Temperature Electrospinning-Fabricated Three-Dimensional Nanofiber Scaffolds for Skin Substitutes
Source: Micromachines (Basel). 2025 Apr 30;16(5):552. doi: 10.3390/mi16050552 (PMC12114171; doi:10.3390/mi16050552)
Supplement: Supplementary file 1 [file micromachines-16-00552-s001.zip › micromachines-3568261-supplementary.pdf]

Supporting Information

Low-Temperature Electrospinning-Fabricated Three-Dimensional Nanofiber Scaffolds for Skin Substitutes

Qiqi Dai [1,2,†], Huazhen Liu [2,†], Wenbin Sun [3], Yi Zhang [3], Weihuang Cai [3], Chunxiang Lu [3], Kaidi Luo [2],
Yuanyuan Liu [2,3,*], Yeping Wang [1,2,4,5,*]

[1] The Third Affiliated Hospital of Shanghai University, Wenzhou 325000, China; qqidia@163.com
[2] School of Medicine, Shanghai University, Shanghai 200444, China; lesyinz@163.com (H.L.); 1849893451@shu.edu.cn (K.L.)
[3] School of Mechatronic Engineering and Automation, Shanghai University, Shanghai 200444, China;
zhangyishu@shu.edu.cn (Y.Z.); cxlu@shu.edu.cn (C.L.)
[4] Wenzhou People's Hospital, Wenzhou 325000, China
[5] Department of Obstetrics and Gynecology, The Third Clinical Institute Affiliated to Wenzhou Medical
University, Wenzhou 325000, China
* Correspondence: yuanyuan_liu@shu.edu.cn (Y.L.); wzwyping@163.com (Y.W.);
Tel.: +86-15900611572 (Y.L.); +86-13587659896 (Y.W.)
† These authors contributed equally to this work.

Table S1: Properties of common materials (including polymers, biomaterials) used for electrospinning

Materials	Young's modulus (MPa)	Melting point (°C)	Water solubility	Common organic solvent	biocompatibility	Ref
PLGA	10–2000	150–200	insoluble	Chloroform<br>DMF<br>THF	Good (degradable, non-toxic)	[22]
PCL	150–600	55–60	insoluble	Acetone<br>DMF<br>THF	Good (slow degradation, low inflammatory response)	[24]
PLA	1000–3500	170–180	insoluble	Chloroform<br>THF<br>DMSO	Good (acidity of degradation products needs to be regulated)	[30]
Collagen	0.1–100	40–60	Partially soluble	Acetic acid<br>HCl	Excellent (promotes cell adhesion and proliferation)	[31]
Chitosan	50–1000	No definite melting point	Partially soluble	Acetic acid<br>Methanol	Good (mild immunogenicity)	[16]
Silk fibroin	100–1000	200–250	insoluble	Methanoic acid<br>HFIP	Excellent (low immunogenicity)	[19]
Mussel protein	/	No definite melting point	insoluble	Methanoic acid<br>HFIP	Non-cytotoxic, low immunogenicity, can promote cell adhesion, proliferation and wound healing	[2]

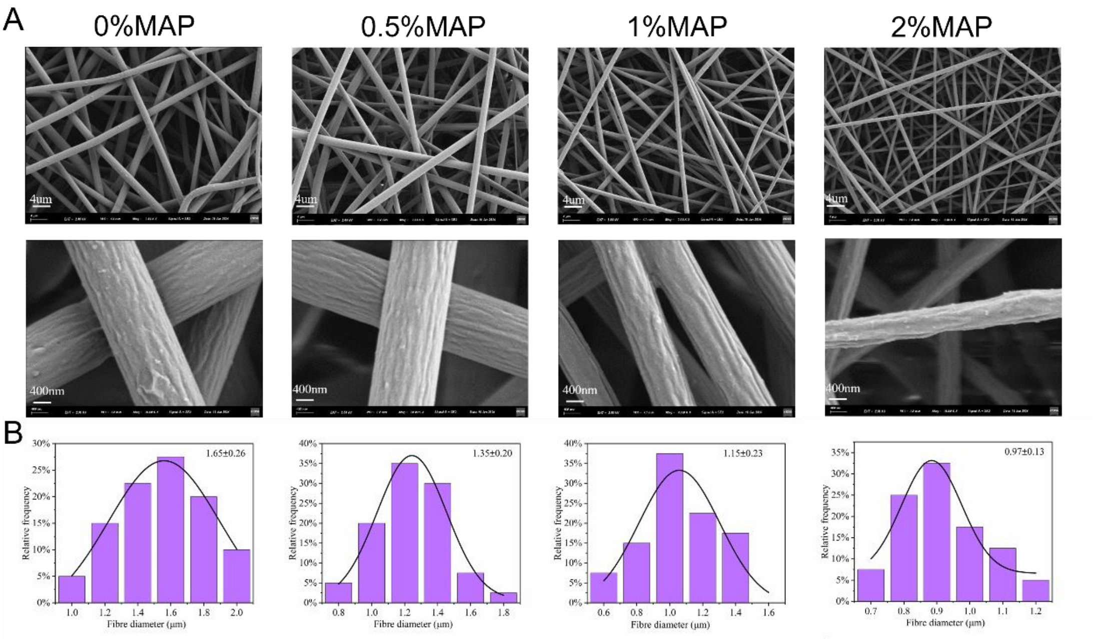

Figure S1.(A) Scanning electron microscopy of PLGA-PCL-0%MAP, PLGA-PCL-0.5%MAP, PLGA-PCL-1%MAP, PLGA-PCL-2%MAP 2D nanofiber scaffolds. (B) Diameter distribution of nanofibers with different MAP concentrations.

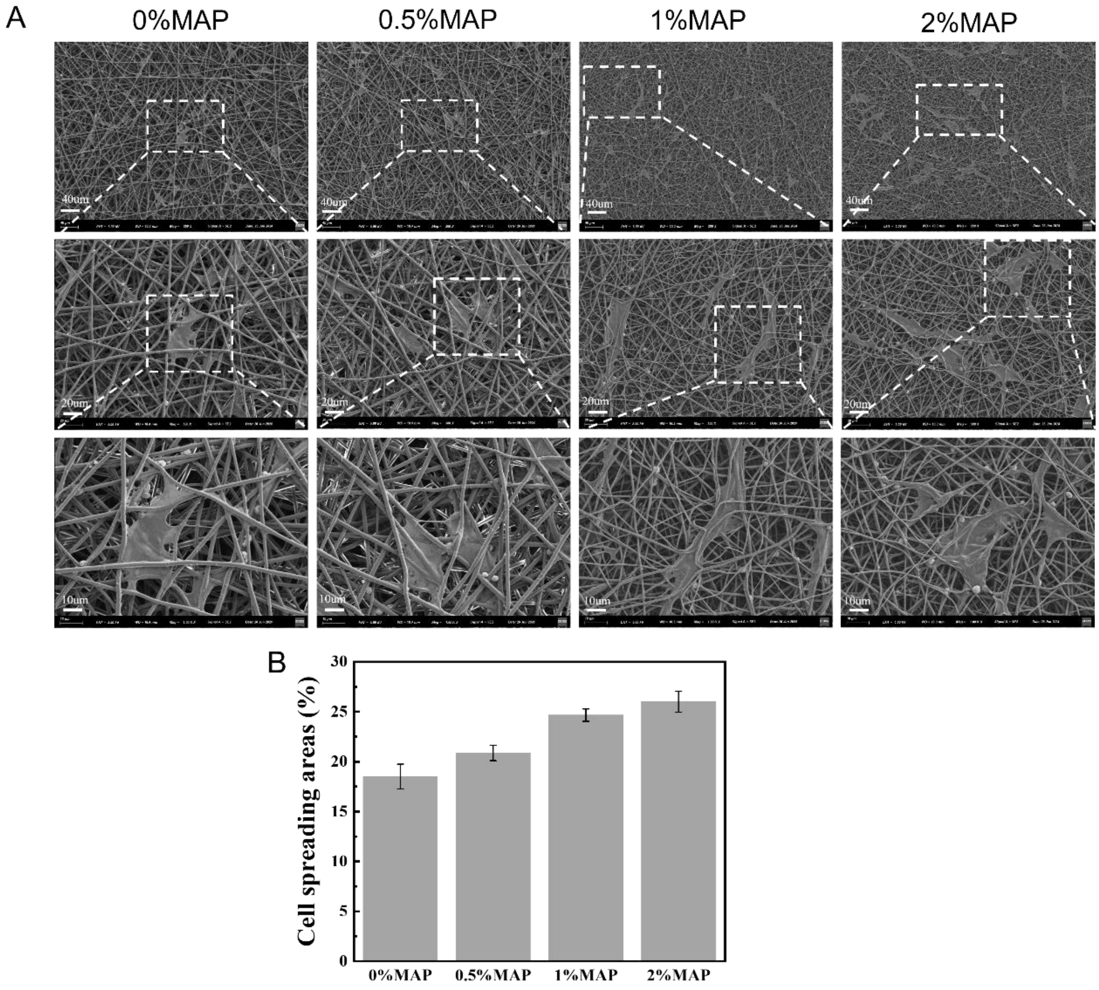

Figure S2. (A)SEM images of HFBs cells inoculated on PLGA-PCL-0%MAP, PLGA-PCL-0.5%MAP, PLGA-PCL-1%MAP, PLGA-PCL-2%MAP nanofiber scaffolds were enlarged by 200, 500 and 1000 times, respectively.(B) Diffusion area of HFBs cells on PLGA-PCL-0%MAP, PLGA-PCL-0.5%MAP, PLGA-PCL-1%MAP, PLGA-PCL-2%MAP nanofiber scaffolds.

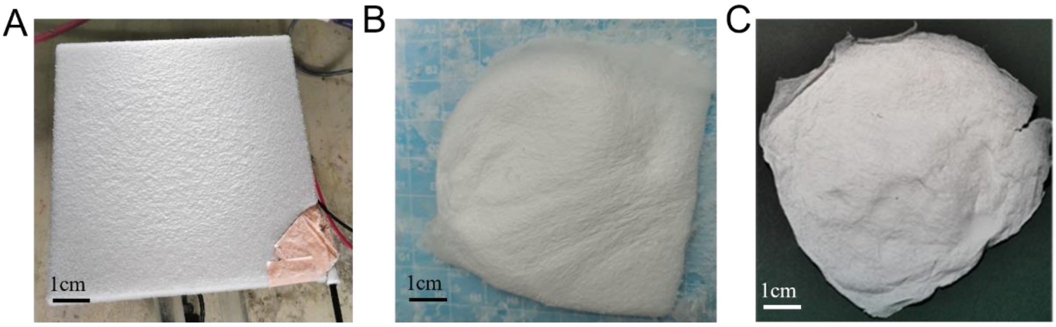

Figure S3. Macrostructure of 3D nanofibers: (A) Fiber deposition on cryo-receiving plate. (B) 3D frozen nanofiber scaffold. (C) 3D dried nanofiber scaffold .

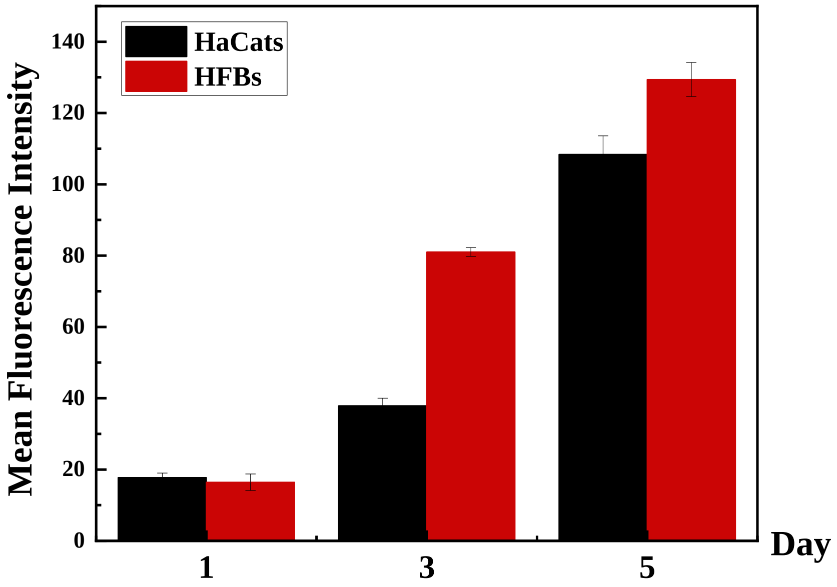

Figure S4. Mean fluorescence intensity of HaCaTs and HFBs cells cultured on 3D nanofiber scaffold for 1, 3 and 5 days.

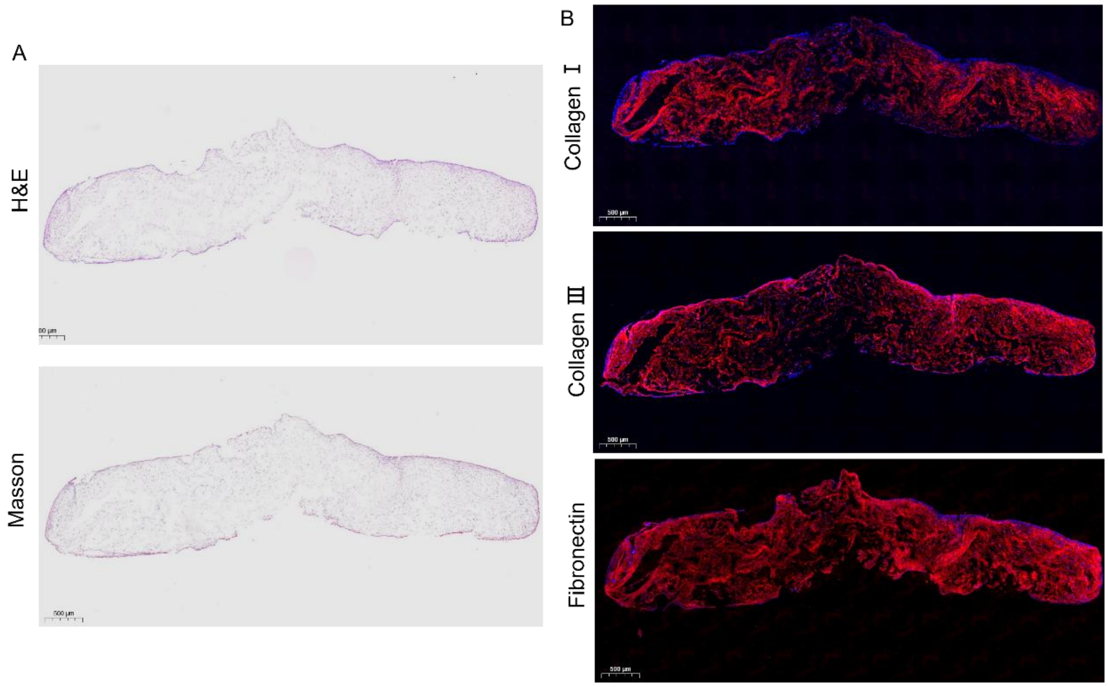

Figure S5. (A) H&E and Masson staining of HFBs cells cultured in vitro on 3D scaffold for one week. (B) Overall images of Col I,Col III, and fibronectin immunofluorescence.

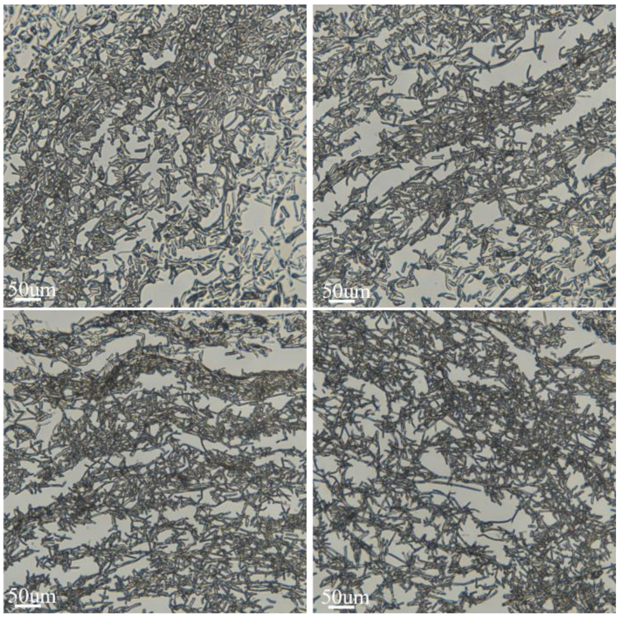

Figure S6. Cross-sectional slice images of 3D nanofiber scaffolds.
